# Correction to: Development and Validation of Arc Nanobodies: New Tools for Probing Arc Dynamics and Function

**DOI:** 10.1007/s11064-022-03589-x

**Published:** 2022-03-31

**Authors:** Yuta Ishizuka, Tadiwos F. Mergiya, Rodolfo Baldinotti, Ju Xu, Erik I. Hallin, Sigurbjörn Markússon, Petri Kursula, Clive R. Bramham

**Affiliations:** 1 Department of Biomedicine, University of Bergen, Jonas Lies 91, 5009 Bergen, Norway; 2 Mohn Center for Research on the Brain, University of Bergen, Bergen, Norway; 3 Faculty of Biochemistry and Molecular Medicine, University of Oulu, Oulu, Finland; 4 Biocenter Oulu, University of Oulu, Oulu, Finland; 5 Department of Pathophysiology and Metabolism, Kawasaki Medical School, 577 Matsushima, Kurashiki, Okayama, 701-0192 Japan

## Correction to: Neurochemical Research 10.1007/s11064-022-03573-5

Unfortunately, after online publication of the article, it has been noticed that there is a typo under the discussion section of the online published article.

Under “Discussion” in para starting “In the parallel study on the generation…” the sentence “Isothermal titration calorimetry showed that ArcNb H11 binds with high affinity (1 nM Kd) and competitively displaces stargazin, an auxiliary subunit of AMPA-type glutamate receptors, which has the highest binding affinity (34.9 nM) of the known Arc NL ligands [33].” should read as “Isothermal titration calorimetry showed that ArcNb H11 binds with high affinity (1 nM Kd) and competitively displaces stargazin, an auxiliary subunit of AMPA-type glutamate receptors, which has the highest binding affinity (34.9 µM) of the known Arc NL ligands [33].” The “µ” symbol wrongly displayed as “n” in the final version of the online publication. This was corrected by publishing this correction article.

