# Supplementary material for: Development and Validation of Arc Nanobodies: New Tools for Probing Arc Dynamics and Function
Source: Neurochem Res. 2022 Mar 20;47(9):2656–66. doi: 10.1007/s11064-022-03573-5 (PMC9463278; doi:10.1007/s11064-022-03573-5)
Supplement: Supplementary file 1 — Supplementary file1 (DOCX 11920 kb) [file 11064_2022_3573_MOESM1_ESM.docx]

**
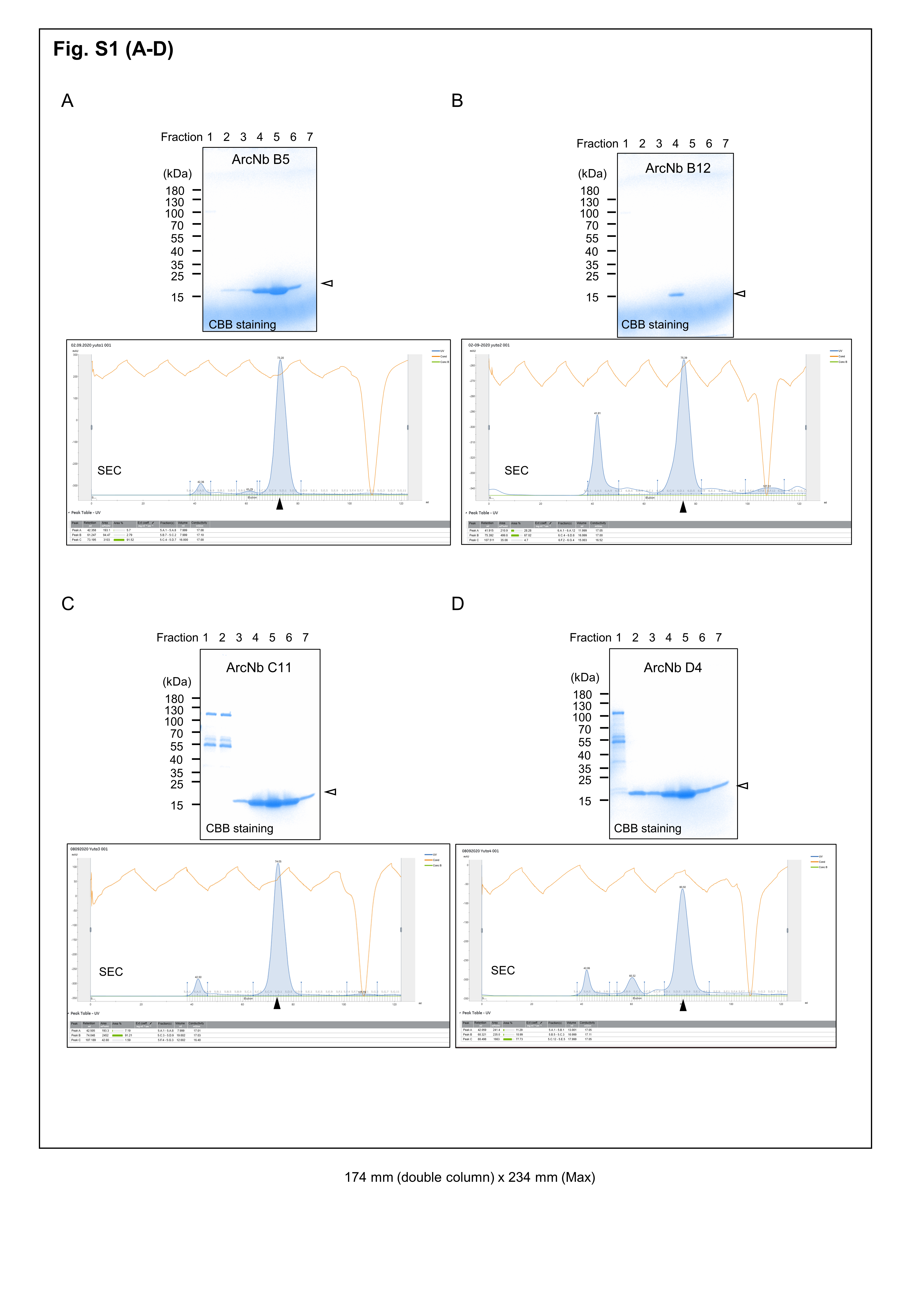

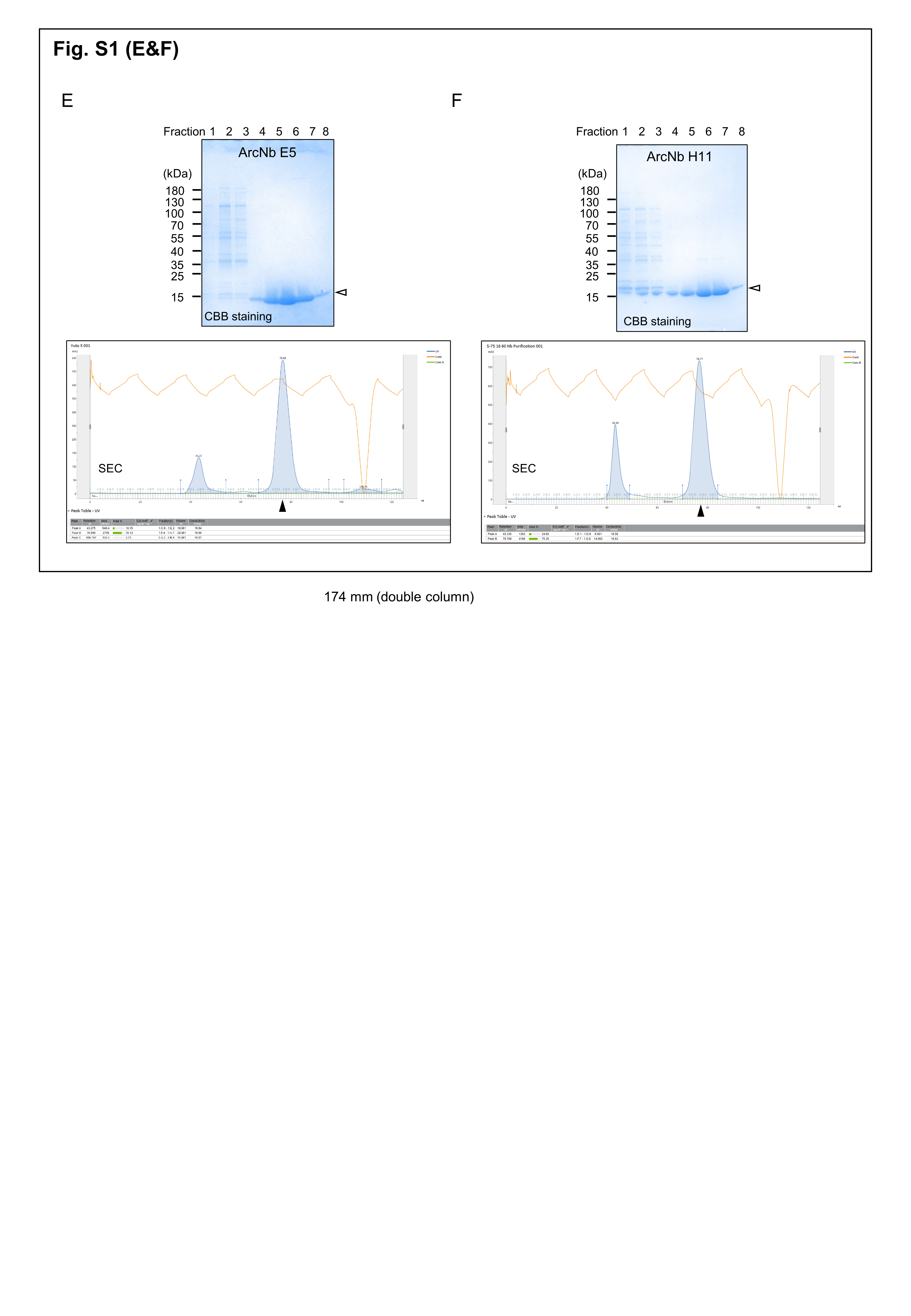

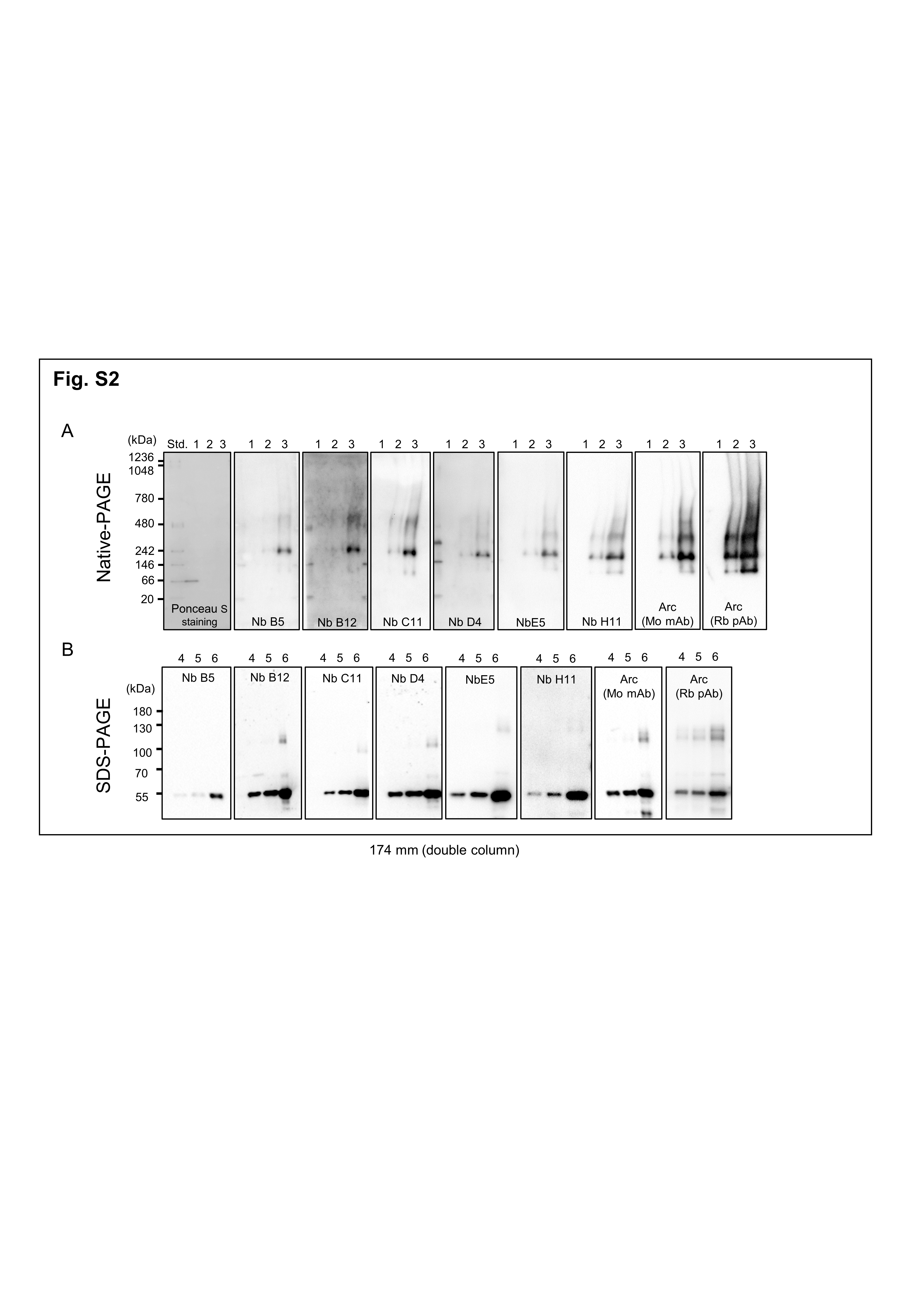
**

**
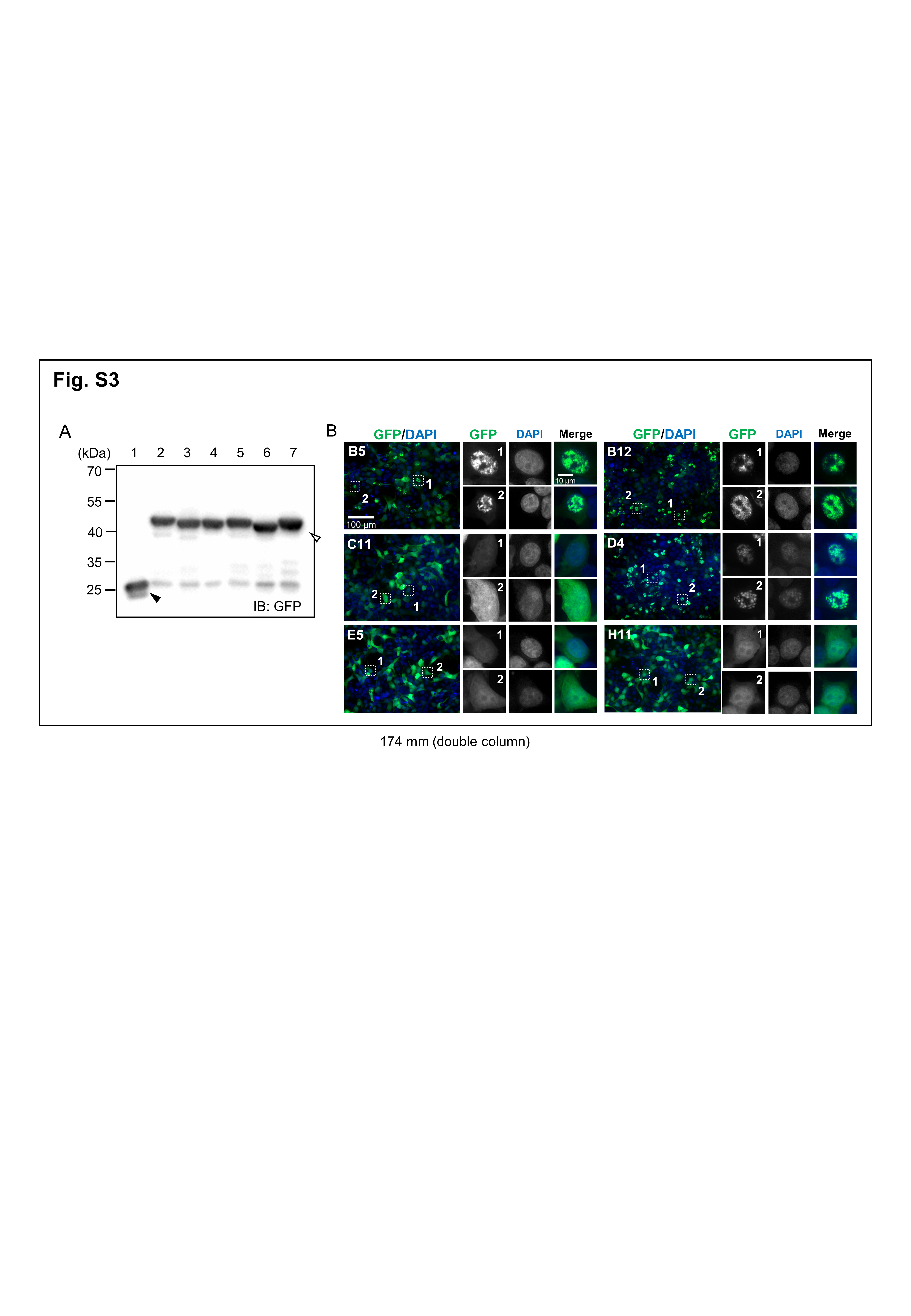

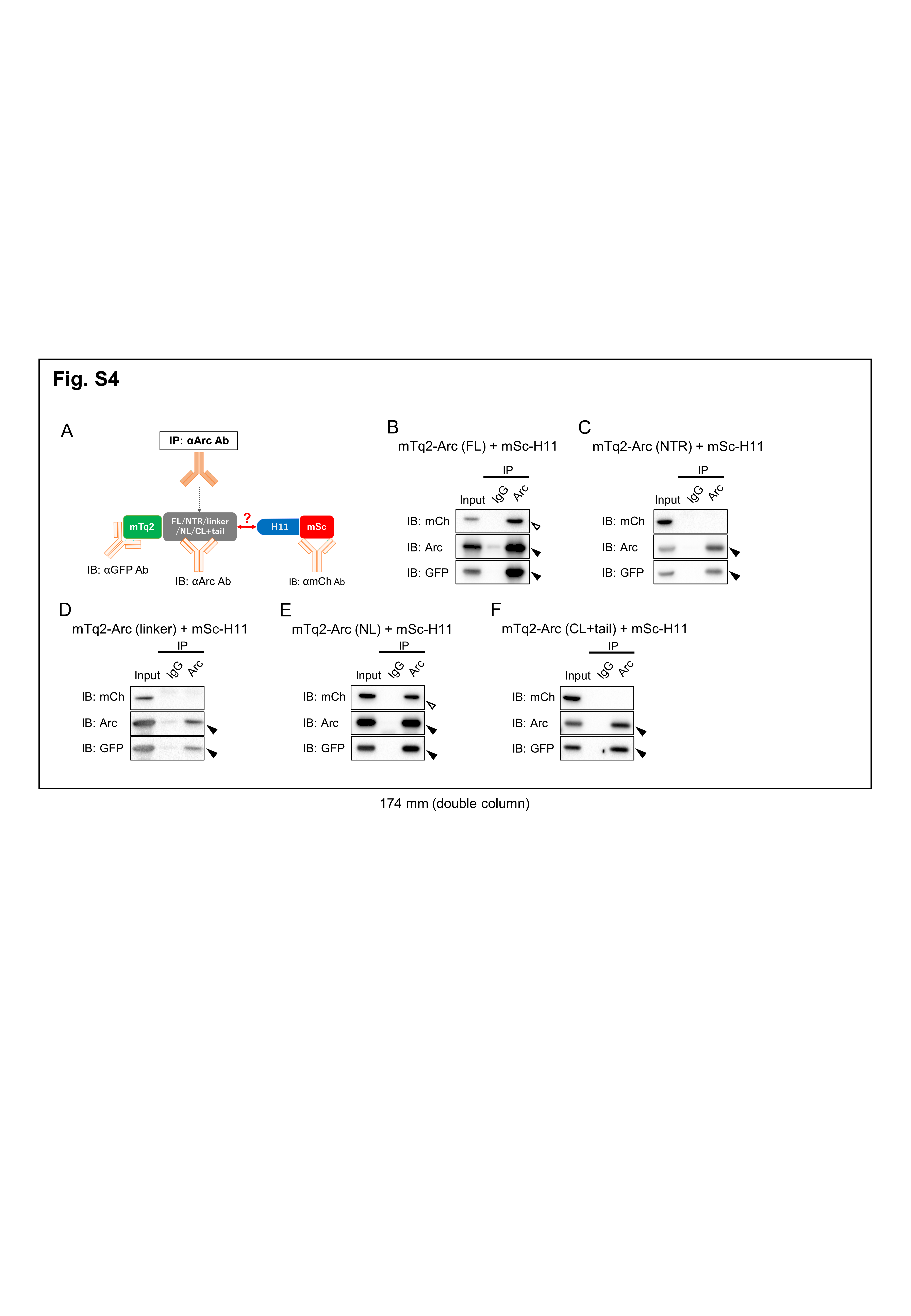
**

**Supplemental Materials**

**Fig. S1.** Quality analysis of purified ALFA-ArcNbs. All purified ALFA-ArcNbs were subjected to SEC followed by SDS-PAGE (***A,*** B5; ***B,*** B12; ***C,*** C11; ***D,*** D4; ***E,*** E5; ***F,*** H11). Upper images are acryl amid gel stained by InstantBlue® Coomassie Protein Stain (Abcam plc, Cambridge, UK) after SDS-PAGE. Open arrowheads indicate purified ALFA-ArcNbs around 15 kDa. Lower images are SEC chromatograms. Closed arrowheads indicate the fractions which were collected

**Fig. S2.** All ArcNbs detect purified Arc recombinant protein both in native and denatured condition. ***A,*** Purified rat Arc mutant protein (Arc^s113-119A^) was subjected to native-PAGE followed by immunoblotting using ALFA-ArcNbs and conventional anti-Arc antibodies. BSA was used as reference for PAGE (~66 kDa in Ponceau S staining). Std., Molecular weight marker; Lane 1, BSA (1 μg); 2, Arc^s113-119A^ (0.1 μg); 3, Arc^s113-119A^ (0.5 μg). ***B,*** Purified Arc^s113-119A^ was subjected to SDS-PAGE followed by immunoblotting. Lane 4, Arc^s113-119A^ (0.05 μg); 5, Arc^s113-119A^ (0.1 μg); 6, Arc^s113-119A^ (0.5 μg)

**Fig. S3.** AcGFP-ArcNb expression in HEK293FT cells as intrabody. ***A,*** HEK293FT cells were transfected with AcGFP control vector (Mock, closed arrowhead) or AcGFP-ArcNb expression vectors (open arrowhead). Cell lysates were subjected to SDS-PAGE followed by immunoblotting using anti-GFP antibody. Lane 1, Mock; 2, B5; 3, B12; 4, C11; 5, D4; 6, E5; 7, H11. ***B,*** HEK293FT cells transfected with AcGFP-ArcNbs were fixed and imaged by fluorescence microscopy. Left, low magnification images with two representative cells marked with a stippled square. Right, high magnification images of the marked cells

**Fig. S4.** Reverse co-IP assay using anti-Arc antibody. ***A,*** Schema of co-IP assay. ***B-F,*** Co-IP assay. HEK293FT cells were co-transfected with mTq2-Arc-FL (***B***), NTR (***C***), linker (***D***), NL (***E***), or CL+tail (***F***) and mSc-H11 constructs. Cell lysates were then subjected to co-IP assay using anti-Arc antibody. Following SDS-PAGE, immunoprecipitants were probed using anti-mCh, Arc, and GFP antibodies. Closed and open arrowheads indicate immunoprecipitated mTq2-Arc variants and co-immunoprecipitated mSc-H11, respectively
